# Efficacy of intravenous N acetylcysteine as an adjuvant therapy in the treatment of acute aluminum phosphide Poisoning: a systematic review and meta-analysis

**DOI:** 10.1186/s40360-023-00699-2

**Published:** 2023-11-03

**Authors:** Heba Othman Shaker, Omar El Sayed Rageh, Maged Alnajar, Nesreen Fares Alshamaly, Walaa Abdelfattah Abdelmaged, Mohamed Abd-ElGawad

**Affiliations:** 1 Supervisor of clinical pharmacy units, Alexandria Main University Hospital, Alexandria, Egypt; 2 Faculty of medicine, Tanta University, Tanta, Egypt; 3 Faculty of pharmacy, Alhikma University, Taiz, Yemen; 4 Faculty of pharmacy and pharmaceutical chemistry, Damascus University, Hama, Syria; 5 Clinical pharmacist, Ministry of health, Alexandria, Egypt; 6 Faculty of medicine, Fayoum University, Fayoum, Egypt

**Keywords:** Aluminum phosphide, N-acetylcysteine, Poisoning, Supportive treatment, Mortality

## Abstract

**Background:**

Aluminum phosphide toxicity is a serious problem in many countries. Unfortunately, there is no specific antidote. N-acetylcysteine has been used in some studies as adjuvant therapy depending on to its antioxidant properties. We hypothesized that IV N-acetylcysteine is effective in reducing mortality rate compared to supportive treatment alone.

**Methods:**

We searched in PubMed, Scopus, Web of Science, and Cochrane Library databases. We only included randomized controlled trials that assessed the efficacy of IV N-acetylcysteine and supportive treatment versus supportive treatment alone in acute aluminum phosphide poisoning. Four investigators independently screened the studies’ results and designed the data extraction sheet. The primary and secondary outcomes were mortality and the need for mechanical ventilation rates. Random effects estimators with weights were used to result in the pooled risk ratios.

**Results:**

We included four randomized controlled trials with 177 patients. 91 patients were distributed in N-acetylcysteine group and 86 patients in the control group. Mortality rates in N-acetylcysteine group and in the control group were 43.95% 66.27% respectively. There was a statistically significant reduction in mortality rate after leave out test (pooled risk ratio, 0.5; 95% confidence interval, 0.32–0.77). Regarding the need for mechanical ventilation, it was measured only in three RCTs. It was assessed in 67 patients in N-acetylcysteine group and 60 patients in the control group. 24 patients were ventilated in N-acetylcysteine group (35.8%) and 29 patients in the control group (48.3%). But it was statistically nonsignificant (pooled risk ratio, 0.71; 95% confidence interval, 0.48–1.04).

**Conclusion:**

Our meta-analysis revealed that IV N-acetylcysteine may be effective in reducing mortality of severe aluminum phosphide poisoning cases.

**Trial registration:**

Registration number in Prospero CRD42022375344 on 25 NOVEMBER 2022, retrospectively registered.

**Supplementary Information:**

The online version contains supplementary material available at 10.1186/s40360-023-00699-2.

## Background

Aluminum phosphide (ALP) or rice tablet is a common pesticide used for agricultural and non-agricultural purposes in Asia and the Middle East. It is highly toxic, cheap, and widely available. In Egypt, it has been noticed that hospital admissions for ALP poisoning are increasing. Many studies have found that mortality rate ranges between 30 and 100% [1]. After consuming aluminum phosphide, the time interval between intake and death is 1–48 h with an average of 3 h [1–3].

The toxicity of ALP appears after contact with water, moisture, or gastric acid (hydrochloric acid), it releases phosphine gas (PH3) which inhibits cytochrome C oxidase, induces oxidative stress, and increases the extra-mitochondrial release of free oxygen radicals resulting in lipid peroxidation and protein denaturation of cellular membranes in different organs. Also, ALP reduces glutathione, which is one of the main antioxidant defenses [2, 3].

The toxicity symptoms usually start with dizziness, nausea, vomiting, retrosternal burning, numbness and rapidly deteriorate to hypotension, shock, respiratory failure, arrhythmias, disseminated intravascular coagulation, hepatotoxicity, and renal failure. Aluminum phosphide poisoning symptoms are non-specific, dose-dependent and the cases deteriorate over time [1, 3].

Unfortunately, there is no specific antidote for ALP. So, all efforts should be made to investigate the effects of drugs that may reduce mortality and improve clinical outcomes. Supportive treatment includes gastric decontamination by early gastric lavage with potassium permanganate, coconut oil, sodium bicarbonate and charcoal administration. Circulatory shock can be treated with fluids resuscitation, vasopressors and corticosteroids .Oxygen is given for hypoxia and patients with respiratory failure need mechanical ventilation support .Inotropes are used in cardiogenic shock and IV sodium bicarbonate in acidosis [1]. Dialysis may be required for severe acidosis and acute renal failure .Considering the induction of oxidative stress, many researchers studied the beneficial effect of many antioxidants as L carnitine, vitamin E ,vitamin C [2].

N-acetylcysteine is widely used as a pharmacological antioxidant and cellular proactive agent against free radicals [4]. N-acetylcysteine replete glutathione reserves by providing cysteine, which is an essential precursor in glutathione production. NAC also binds to the toxic metabolites and scavenges free radicals. It increases oxygen delivery to tissues, increases mitochondrial ATP production, and alters the microvascular tone to increase the blood flow and oxygen delivery to the liver and other vital organs [5], and in animal studies NAC has been shown the ability to reduce myocardial oxidative injury [6], so according to these features, it can have a significant effect on ALP poison.

Many studies have investigated the effect of NAC in the treatment of ALP [7–10]. In this systematic review and meta-analysis, we hypothesized that IV NAC as adjuvant therapy can reduce the mortality rate of acute aluminum phosphide poisoning.

## Methods

### Study design

This study’s meta-analysis followed the PRISMA guidelines and the Cochrane Handbook of Systematic Reviews of Interventions. Supplementary file 1 (PRISMA checklist 2020).

### Literature search

PubMed, Cochrane Central Register of Controlled Trials (CENTRAL), Scopus, and Web of Science were utilized for the search of articles published from January 1973 to January 2022. The data collection process took place on 15 September of 2022. The search strategy used can be found in supplementary file 2.

### Eligibility criteria & study selection

Firstly, we included randomized controlled trials (RCTs), case control, cohort, and cross-sectional studies, the main goal was to compare the response of patients poisoned with aluminum phosphide to adjuvant therapy with IV N-acetylcysteine versus supportive treatment alone to assess multiple outcomes that will be discussed later.

Our exclusion criteria were unreliable or insufficient data for extraction, reviews, book chapters, theses, editorials, letters, conference papers, and non-English studies. Animal, in vitro studies, case reports, non-clinical studies, literature reviews, and meta-analyses were excluded. Studies on mild to moderate ALP cases were excluded, as were studies on nonclinical outcomes.

Four reliable authors screened the studies by title, abstract, and full text on an excel sheet for eligibility. The team leader resolved any disagreements. 6 studies were included after eligibility assessment. Due to the low number of studies with small sample size of patients, the decision was to include only RCTs which have more valid design. Two studies (a case control and a cohort) were excluded.

### Quality assessment

Four authors used Cochrane risk-of-bias tool for randomized trials (ROB1) to assess the quality of the selected studies. The ROB1 tool consists of six domains: the randomization process, deviations from the intended interventions, missing outcome data, measurement of the outcome, selection of the reported result, and other biases. The evaluators’ responses were categorized as: low risk, high risk, unclear risk of bias.

### Data extraction and study outcomes

Four reliable authors/reviewers performed the data extraction in a well-defined excel sheet, the excel sheet items were categorized as: general information about the study designs, participants, and intervention, baseline information about certain parameters in the study as: Age, sex, heart rate, and systolic blood pressure, enzymes as malondialdehyde (MDA) and total antioxidant capacity (TAC), cardiac enzymes as creatine kinase MB (CKMB), and the outcome information including mortality rate, and the need for mechanical ventilation and hospitalization time.

We selected mortality rate and mechanical ventilation for the meta-analysis as they were measured in at least three RCTs.

### Outcome definition

Our primary outcome was to measure mortality rate among patients in the intervention and control arms by identifying the number of people who survived and who died. The secondary outcome was to.

evaluate the need for mechanical ventilation by identifying the number of people who ventilated and did not ventilate.

### Data synthesis and assessment of heterogeneity

We performed all statistical analyses using R software Version 4.3.0. The present meta-analysis estimated the pooled risk ratio (RR) for dichotomous data, with 95% confidence intervals (CI). The significance level was set at a P-value less than 0.05.

We assessed the heterogeneity using the I-square and p-value. The analysis was considered heterogeneous if it had a p-value less than 0.05 or an I-square less than 50%. A random-effect model was applied if heterogeneity was detected, and a leave one out test was performed to determine which study was causing the heterogeneity.

## Results

### Data collection and study selection

**Fig. 1:**
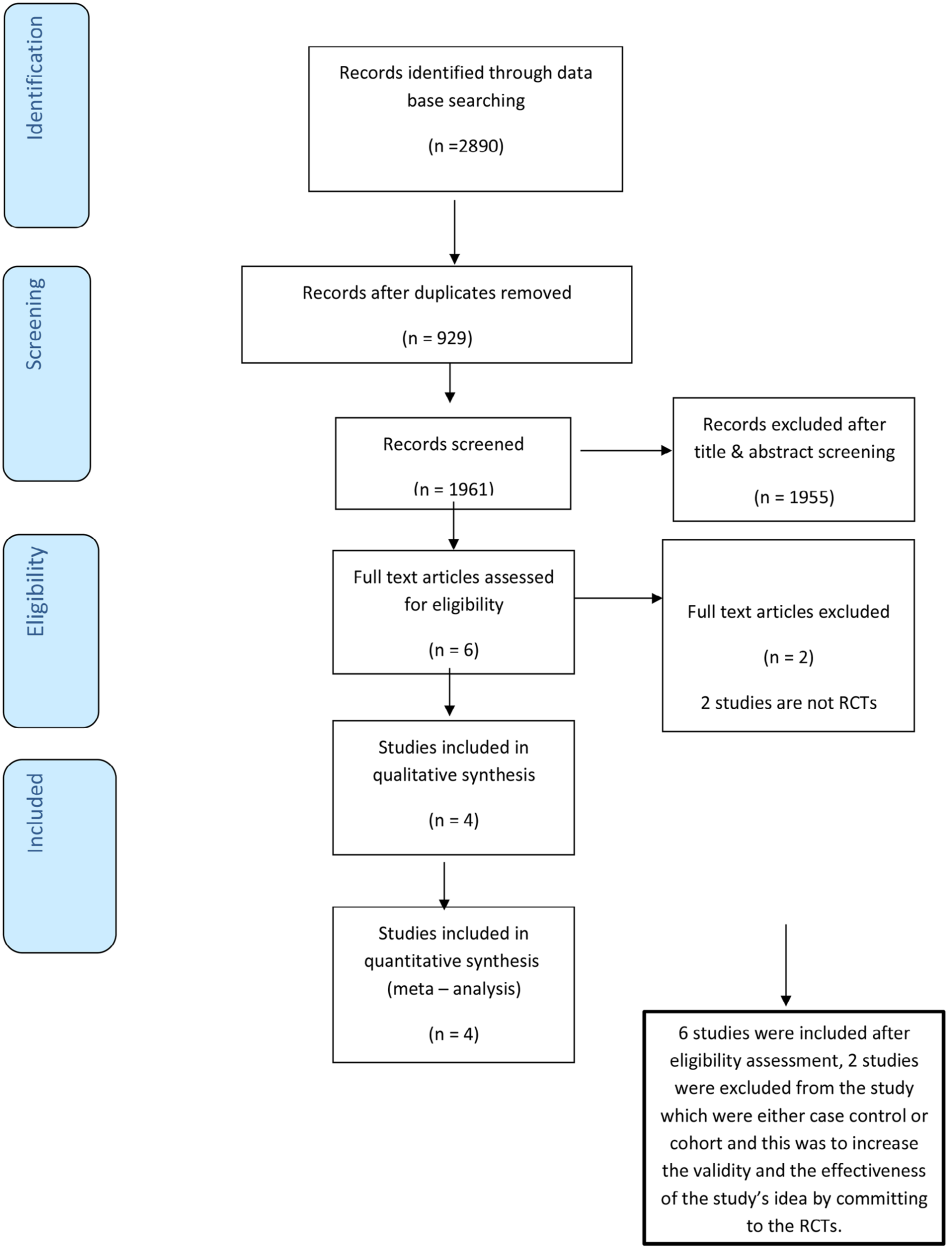
PRISMA figure

Our search retrieved 2890 records from PubMed, Scopus, Web of Science, and Cochrane library. There were 929 duplicates. After title and abstract screening, we eliminated 1955 records. Afterward, we screened 6 full texts for eligibility, two studies were excluded because they are not RCTs. Finally, four records were included in our study: All four studies were included in the meta-analysis PRISMA figure (Fig. 1). We analyzed 177 patients from the four RCTs (Studies’ summary and patients’ characteristics were presented in Table 1). 91 and 86 patients were distributed to the intervention and control group respectively.

**Table 1:** The summary of included studies and baseline characteristics of participants

Study	Country	Number of patients	Inclusion criteria	Intervention	Age	Sex	Systolic blood pressure	Heart rate	MDA	TAC	CKMB	Mortality rate	Ventillation rate	Hospitalization time						
Tehrani, H. et al. 2013	Iran	37(22 in intervention and 15 in control)	Acute aluminum phosphide-intoxicated patients with no history of diabetes mellitus, cardiovascular, respiratory, renal and hepatic failure, and no medical management for aluminum phosphide poisoning in any medical center before. admission	NAC (140 mg/Kg/IV infusion as a loading dose and 70 mg/Kg/IV infusion every 4 h up to 17 doses)	23.5 ± 7.8 24.7 ± 6.4	M/F29.7%/29.7% M/F21.6%/18.9%	93.7 ± 17.8 87.3 ± 13.6	88.7 ± 17.1 94.6 ± 21.1	On admission: 195.7 ± 67.4 139 ± 28.2 24 h after admission 174.6 ± 48.9 149.6 ± 35.2	On admission 11.4 ± 2.2 13.3 ± 2.5 24 h after admission 12.8 ± 5.7 17.3 ± 5.6	NA NA	Intervention group: 36% (8 out of 22) Control group: 60% (9 out of 15)	Intervention group: 45.4% (10 out of 22) Control group: 73.3% (11 out of 15)	2.7 ± 1.8 days 8.5 ± 8.2 days						
Bhalla A. et al. 2017	India	50(24 in intervention and 26 in control)	All the patients presenting to emergency outpatient department with alleged history of ingestion of ALP was screened and those. with shock were eligible	NAC in the dose of 150 mg/kg intravenous over 1 h, followed by 50 mg/kg over 4 h, followed by 100 mg/kg 16 h in 5% dextrose	< 30 years:24% 40% > 30 years:24% 12%	M/F38%/10% M/F32%/20%	77.52 ± 8.59 74.15 ± 9.2	134.015 ± 14.2 137.91 ± 16.99	NA NA	NA NA	NA NA	Intervention group: 87.5% (21 out of 24) Control group: 88.5% (23 out of 26)	Data not available.							
Emam et al. − 2020	Egypt	60 (30 in intervention and 30in control)	Subjects (aged 12 years or older) with symptomatic acute ALP poisoning and a history of rice tablets ingestion presented within 6 h from exposure with no previous medical treatment for phosphide intoxication in any medical center prior to admission were included	Dose 1: 150 mg/kg IV, mixed in 200 mL of D5W and infused over 1 h. Dose 2: 50 mg/kg IV, mixed in 500 mL D5W and infused over 4 h. Dose 3: 100 mg/kg IV, mixed in 1000 mL. D5W and infused over 16 h.	24.4 ± 10.5 24.4 ± 9.66	M/F18.3%/31.6% M/F18.3%/31.6%	92.16 ± 21.9 94.83 ± 21.1	104.57 ± 20.5 104.27 ± 20.5	NA NA	NA NA	NA NA	Intervention group: 20% (6 out of 30) Control group: 43.3% (13 out of 30)	Intervention group: 23.3% (7 out of 30) Control group: 20% (6 out of 30)							
El-Ebiary et al. − 2017	Egypt	30 (15 in intervention and 15 in control)	Patients (male or female, aged 18 years or older) with symptomatic acute ALP poisoning (deliberate or accidental), were included	Patients received NAC 140 mg/Kg IV infusion (as a loading dose), then 70 mg/Kg IV infusion every 4 h up to 17 doses, In addition, the routine treatment was given, and it consists of patient resuscitation, gastric decontamination	From 18–28 years old : (86.7%) (80.0%) From 28–38 years old : (13.3%) (6.7%) From 38–48 years old: (0.0%) (13.3%)	M/F23.3%/26.6% M/F20%/30%	Hypotension (73.3%)(93.3%) Hypertension (6.7%)(0.0%) Normal (20.0%)(6.7%)	Undetected (0.0%) (13.3%) Bradycardia (0.0%) (6.7%) Tachycardia (33.3%) (46.7%) Normal (66.7%) (33.3%)	On admission 17.48 ± 7.48 16.23 ± 7.91 After treatment 2.79 ± 1.82 17.26 ± 6.39 -	On admission 1.99 ± 0.77 3.23 ± 2.16 After treatment 0.66 ± 0.26 2.15 ± 1.44	NA NA	Intervention group: 33.3% (5 out of 15) Control group: 80% (12 out of 15)	Intervention group: 46.7% (7 out of 15) Control group: 80% (12 out of 15)	48 (13–48) 12 (8–13)						

NAC: N-acetyl cysteine

SD: standard deviation

IQR: Interquartile range

M: male

F: female

CKMB: creatine kinase myocardial band

NA: Not Applicable

TAC: Total Antioxidant Capacity of plasma

MDA: Malondialdehyde

Numbers are expressed as : mean ± standard deviation, median (interquartile range) or percentage

Considering the low sample size, we did not do subgroup analysis to find a relation between IV N-acetylcysteine and patients ’characteristics. Generally, among the total study group, 50.28% were males (27.11% in NAC group, 23.16% in placebo) and 49.7% were females (24.29% in NAC group,

25.42% in placebo). Majority of our patients were in the second and third decades of life. Regarding the vital signs, most of patients had tachycardia. The systolic blood pressure values were either in or below the normal range.

### Outcome measures

#### Mortality rate

Results showed that 40 patients died in the NAC group (43.95%) and 57 patients died in the control group (66.27%). Pooled data were heterogeneous (80%heterogenity) under a random effect model. The heterogeneity was best resolved by leaving out test under a random effect model (p = 0.76, I² = 0%).

**Fig. 2:**
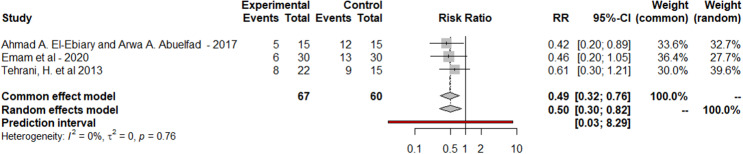
The forest plot of the mortality rate (after leaving out test)

There was a statistically significant reduction in mortality rate (pooled risk ratio, 0.5; 95% confidence interval, 0.32–0.77) p value = 0.002 as was presented in Fig. 2.

### Need for mechanical ventilation

**Fig. 3:**
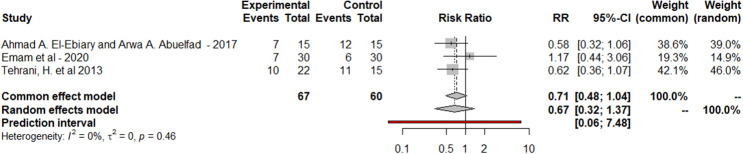
The forest plot of mechanical ventilation rate

Regarding the need for mechanical ventilation as a secondary outcome, it was measured only in three RCTs. It was assessed in 67 patients in the NAC group and 60 patients in the control group. It showed that 24 patients ventilated in the NAC group (35.8%) and 29 patients ventilated in the control group (48.3%). Pooled data were homogenous under a fixed effect model (p = 0.43, I² = 0%), But the reduction in the rate of mechanical ventilation in the intervention group was statistically nonsignificant (pooled risk ratio, 0.71; 95% confidence interval, 0.48–1.04). p value = 0.08 as was presented in Fig. 3.

### Hospitalization time

This secondary outcome was only measured in two studies. First study Tehrani, H. et al. − 2013 reported that the duration of hospitalization was significantly decreased in NAC group in comparison with control group (2.7 ± 1.8 days vs. 8.5 ± 8.2 days, p value = 0.02), and second study El-Ebiary et al.

– 2017 reported that the hospitalization time showed statistically significant differences between NAC group and control group with median 48 h and 12 h respectively, and p value = 0.013.

We didn’t do a meta-analysis for this outcome because it was measured in those two studies only.

### Serum levels of MDA

This secondary outcome only measured in two studies. First study Tehrani, H. et al. – 2013 reported that on admission time, the mean values of MDA serum levels were 195.7 ± 67.4 and 139 ± 28.2 µmol/l in NAC group and control group respectively with statistically significant difference between the two groups (p value = 0.005). However, 24 h after admission the mean value of MDA serum levels were 174.6 ± 48.9 and 149.6 ± 35.2 µmol/l in groups NAC and control respectively with statistically significant difference noticed between the two groups (*p value* = 0.03).

And second study El-Ebiary et al. – 2017 reported that on admission time, the mean values of MDA serum levels were 17.48 ± 7.48 and 16.23 ± 7.91 nmol/ml in NAC group and control group respectively with no statistically significant difference between the two groups (p value = 0.660). However, 24 h after admission the mean values of MDA serum levels were 2.79 ± 1.82 and 17.26 ± 6.39 nmol/ml in groups NAC and control respectively with statistically significant difference between the two groups (*p value* < 0.001).

A meta-analysis could not be done because it was measured in only those two studies.

### Serum levels of TAC

This secondary outcome only measured in two studies. First study Tehrani, H. et al. – 2013 reported that on admission time, the mean values of TAC serum levels were 11.4 ± 2.2 and 13.3 ± 2.5 mmol/l in groups NAC and control respectively with significant difference between the two groups (p value = 0.03). However, 24 h after admission the mean value of TAC serum levels were 12.8 ± 5.7 and 17.3 ± 5.6 mmol/l in groups NAC and control respectively, with no statistically significant difference between the two groups (*p value* = 0.65).

And second study El-Ebiary et al. – 2017 reported that on admission time, the mean values of TAC serum levels 1.99 ± 0.77 and 3.23 ± 2.16 mmol/l in groups NAC and control respectively with no significant difference between the two groups ( p value = 0.052). However, 24 h after admission the mean value of TAC serum levels were 0.66 ± 0.26 and 2.15 ± 1.44 mmol/l in groups NAC and control respectively, with statistically significant difference between the two groups (*p value* = 0.001).

A meta-analysis for this outcome is inapplicable as it was measured in only those two studies.

**Fig. 4:**
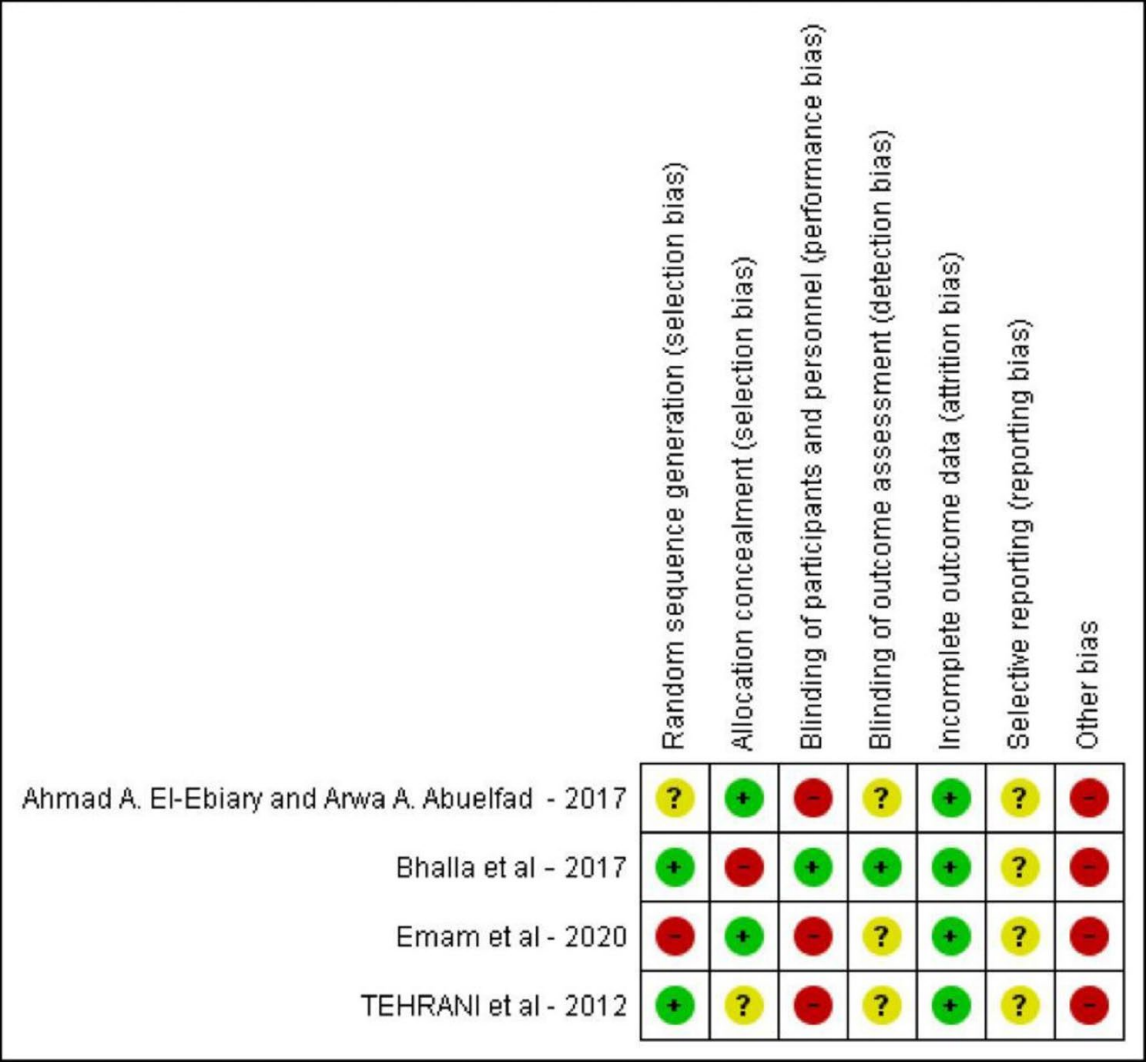
The studies’ risk of bias figure

**Fig. 5:**
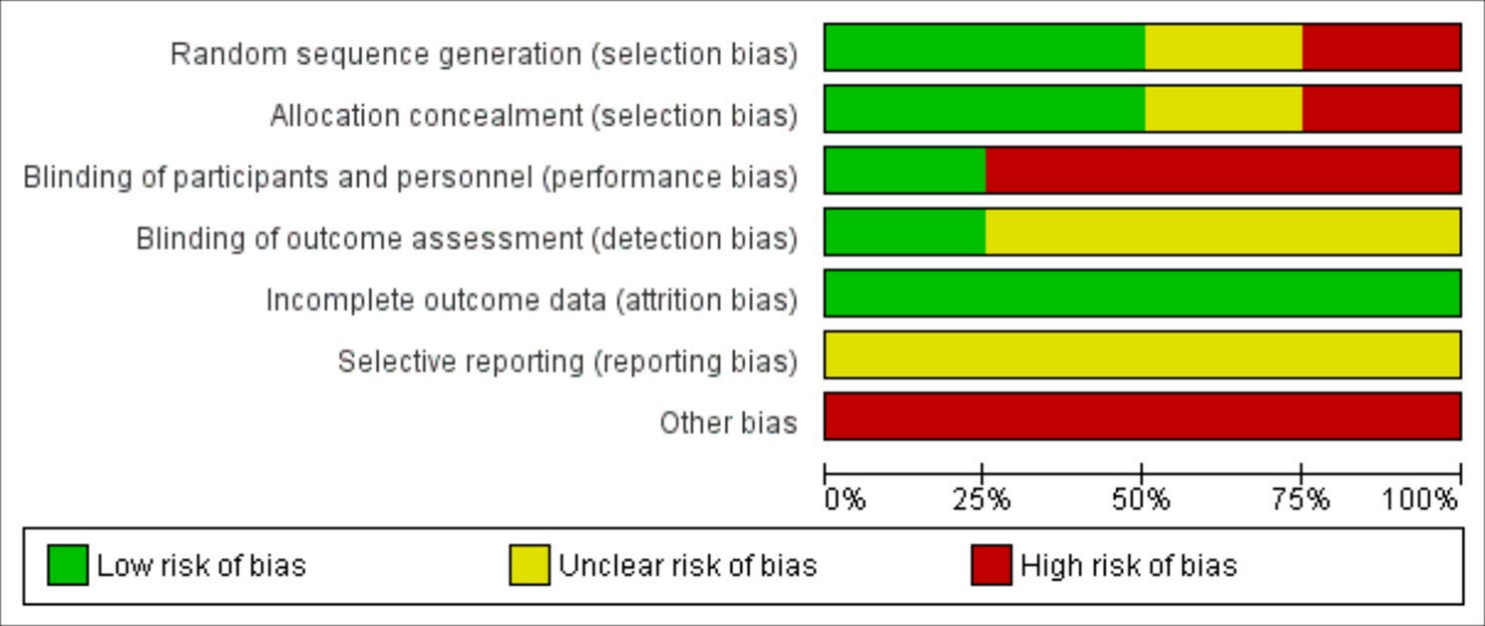
The studies’ risk of bias graph

Quality assessment results:

The risk of bias summary has been classified according to ROB 1 in Figs. 4 and 5.

There was a high risk of bias of blinding of participants and personnel in three studies (El Ebiary et al., Bahall et al. and Tehrani et al.).All the studies analyzed the results according to intention to treat analysis.The studies ’protocols were unavailable, so we considered the risk of selective reporting unclear .The four studies had the same limitation of a small sample size.

## Discussion

ALP toxicity is a serious problem in many countries. Till now there is no specific antidote and patients are treated with supportive treatment only. Finding alternative treatment modalities is crucial.

There are some reviews that discuss the epidemiological, toxicological, and clinical/pathological aspects of ALP poisoning and its treatment [1,2 ]. The mechanism of ALP toxicity involves the suppression of oxidative phosphorylation and the cytochrome-c oxidase enzyme, which results in the failure of cellular respiration and induces oxidative stress that damages cells by causing lipid.

peroxidation, denaturation of cell membrane proteins, and hypoxic damage [3 ]. So, antioxidant therapy might have a therapeutic benefit in acute ALP intoxication.

In this meta-analysis, all the included studies assessed the effect of IV NAC as an adjuvant therapy compared to supportive treatment alone [7–10 ]. The analysis revealed that IV NAC can reduce mortality rate. After the exclusion of Bhalla A. et al. 2017 in the leave out test of the mortality rate, the heterogeneity was resolved, and the results were statistically significant.

The mortality rates published separately in three studies showed significant reduction in NAC treated group. (Emam et al. 2020, El ebiary et al. 2017, Tehrani et al. 2012). In (Bhalla et al. 2017) there was no significant difference in its analysis [7,9–10 ].

Only three studies measured the need for mechanical ventilation (Tehrani, H. et al. 2013, Emam et al. 2020 and El-Ebiary et al. − 2017). Although the results of the meta-analysis showed a reduction in NAC arm, it was statistically non-significant [7,9–10 ].

We cannot assess the duration of hospitalization in a meta-analysis as it was measured in Tehrani, H. et al. 2013 and El-Ebiary et al. − 2017 only [7, 10 ].

The oxidative stress process and the consumption of reduced glutathione cause TAC to rise. Based on these facts, elevated serum levels of MDA and TAC in ALP intoxicated patients compared to the reference values were seen as indicators for ALP-induced oxidative stress.

According to (El ebiary et al. 2017, Tehrani et al. 2012), results pointed to a statistically significant reduction in serum levels of MDA and TAC in ALP intoxicated patients after NAC treatment. But we cannot analyze them as outcomes as they were assessed in two studies only [7, 10 ].

Taghaddosinejad et al. 2016 is a case control study of 63 patients. It showed that adjuvant IV NAC therapy had a protective effect against cardiovascular complications in ALP poisoned patients. CK-.

MB, Creatine phosphokinase (*CPK*), heart rate, and systolic blood pressure means became significantly different over time (0, 12, 18, and 24 h) in both groups (P < 0.001) [11].

Agrawl et al. 2014 cohort study determined the serum level of antioxidant enzymes (viz. catalase, superoxide dismutase (SOD) and glutathione reductase (GR)) and found that the baseline level of catalase and SOD were significantly reduced. Baseline GR level was not suppressed but rather increased with due time, and more so in the treatment group. Also, NAC along with supportive treatment improved survival [12].

### The strengths of this meta-analysis

As far as we know, no previous meta-analysis on this topic has been published. We searched in four mega database sites for data collection, all the included studies were RCTs as we excluded other types.

All the studies analyzed the results according to intention to treat analysis and finally our meta-analysis measured the mortality rate and the need for mechanical ventilation which are two important outcomes in ALP toxicity.

### The limitations

The most obvious limitation is the small sample size and small number of included studies (177 patients from four RCTs) .As mentioned in the methodology section that after eligibility assessment six studies were included. As a result of low number of studies and small number of patients, the decision was to include only RCTs which have more valid design. Both case control and cohort studies were excluded. The heterogeneity is considered to be due to high risk of bias and variability in.

Some outcomes. The observed heterogeneity in pooled data concerning mortality rate was successfully treated through a leave-one-out test.

## Conclusion

Our meta-analysis showed that IV administration of NAC along with supportive treatment for severe cases of ALP poisoning admitted to hospitals has a statistically significant lower risk of mortality than supportive treatment alone. But no statistically significant difference has been proven in the need for mechanical ventilation.

Further studies should be done on a larger number of patients and on other clinical or biochemical outcomes.

### Electronic supplementary material

Below is the link to the electronic supplementary material.

Supplementary file 1: (PRISMA checklist 2020)

Supplementary file 2: (The search strategy)

## Data Availability

The datasets used and/or analyzed during the current study are available from the corresponding author on reasonable request.

## Abbreviations

IV: Intravenously
ALP: Aluminum phosphide
NAC: N-acetylcysteine
RCTs: Randomized controlled trials
ROB: Cochrane risk-of-bias tool for randomized trials
MDA: Malondialdehyde
TAC: Total antioxidant capacity
CKMB: Creatine kinase MB
RR: Risk ratio
CI: Confidence intervals
SOD: Superoxide dismutase
GR: Glutathione reductase
CPK: Creatine phosphokinase
