# Supplementary material for: Efficacy of intravenous N acetylcysteine as an adjuvant therapy in the treatment of acute aluminum phosphide Poisoning: a systematic review and meta-analysis
Source: BMC Pharmacol Toxicol. 2023 Nov 3;24:59. doi: 10.1186/s40360-023-00699-2 (PMC10625249; doi:10.1186/s40360-023-00699-2)
Supplement: Supplementary file 2 — (The search strategy) [file 40360_2023_699_MOESM2_ESM.docx]

**Supplementary file (2):**

**Search strategy**

**We searched for Aluminum phosphide poisoning and N- acetyl cysteine.**

**PubMed:**

(Acetylcysteine OR acetadote OR hidonac OR fluimucil OR Parvolex OR solumucol [Mesh] OR acemuc [Mesh] OR " Bisolvon NAC "[Mesh] OR NALC OR "N-acetyl- l-cysteine" OR "N-acetylcysteine" OR "N- Acetyl Cysteine" OR "N-Acetylcysteinamide" OR "L-acetylcysteine" OR NAC OR "acetyl –cysteine" OR “acetyl cysteine” OR acetylcysteine) AND ("Aluminium Phosphide" OR phosphines OR "aluminum phosphide"[All Fields] OR AIP OR AP OR AlPO4 OR alumanylidynephosphane OR "Phostoxin [Mesh]" OR Fumitoxin OR "quickphos [Mesh]" OR "Celphos [Mesh]" OR Phostek OR Celphine OR Celphide OR "Aluminium fosfide" OR “Detia gas Ex-B” OR Delicia OR "Fosfuri di alluminio" OR “Delicia gastoxin [Mesh] "OR "rice tablet")

Fields: All fields

Limitations: no limitations applied

Date of search: 15/9/2022

Number of results: 687 results

**Cochrane:**

1. Acetylcysteine OR acetadote OR hidonac OR fluimucil OR Parvolex OR NALC OR N-acetyl- l-cysteine OR N-acetylcysteine OR N- Acetyl Cysteine OR N-Acetylcysteinamide OR L-acetylcysteine OR NAC OR acetyl –cysteine OR acetyl cysteine OR acetylcysteine
2. Aluminium Phosphide OR phosphines OR "aluminum phosphide" OR AIP OR AP OR AlPO4 OR alumanylidynephosphane OR Fumitoxin OR Phostek OR Celphine OR Celphide OR "Aluminium fosfide" OR “Detia gas Ex-B” OR Delicia OR Fosfuri di alluminio OR rice tablet
3. #1 AND #2

Fields: All fields

Limitations: no limitations applied

Date of search: 15/9/2022

Number of results: 29 results

**Scopus:**

ALL((Acetylcysteine OR acetadote OR hidonac OR fluimucil OR Parvolex OR NALC OR "N-acetyl- l-cysteine" OR "N-acetylcysteine" OR "N- Acetyl Cysteine" OR "N-Acetylcysteinamide" OR "L-acetylcysteine" OR NAC OR "acetyl –cysteine" OR “acetyl cysteine” OR acetylcysteine) AND ("Aluminium Phosphide" OR phosphines OR "aluminum phosphide" OR AIP OR AP OR AlPO4 OR alumanylidynephosphane OR Fumitoxin OR Phostek OR Celphine OR Celphide OR "Aluminium fosfide" OR “Detia gas Ex-B” OR Delicia OR "Fosfuri di alluminio" OR "rice tablet"))

Fields: All fields

Limitations: no limitations applied

Date of search: 15/9/2022

Number of results: 740 results

**Web of Science:**

ALL=((Acetylcysteine OR acetadote OR hidonac OR fluimucil OR Parvolex OR NALC OR "N-acetyl- l-cysteine" OR "N-acetylcysteine" OR "N- Acetyl Cysteine" OR "N-Acetylcysteinamide" OR "L-acetylcysteine" OR NAC OR "acetyl –cysteine" OR “acetyl cysteine” OR acetylcysteine) AND ("Aluminium Phosphide" OR phosphines OR "aluminum phosphide" OR AIP OR AP OR AlPO4 OR alumanylidynephosphane OR Fumitoxin OR Phostek OR Celphine OR Celphide OR "Aluminium fosfide" OR “Detia gas Ex-B” OR Delicia OR "Fosfuri di alluminio" OR "rice tablet"))

Fields: All fields

Limitations: no limitations applied

Date of search: 15/9/2022

Number of results: 1433 results
